# Secondary migration and leakage of methane from a major tight-gas system

**DOI:** 10.1038/ncomms13614

**Published:** 2016-11-22

**Authors:** James M. Wood, Hamed Sanei

**Affiliations:** 1 Encana Corporation, 500 Centre Street SE, Calgary, Alberta, Canada T2P 2S5; 2 Geological Survey of Canada, 3303 33^rd^ Street NW, Calgary, Alberta, Canada T2L 2A7

## Abstract

Tight-gas and shale-gas systems can undergo significant depressurization during basin uplift and erosion of overburden due primarily to the natural leakage of hydrocarbon fluids. To date, geologic factors governing hydrocarbon leakage from such systems are poorly documented and understood. Here we show, in a study of produced natural gas from 1,907 petroleum wells drilled into a Triassic tight-gas system in western Canada, that hydrocarbon fluid loss is focused along distinct curvilinear pathways controlled by stratigraphic trends with superior matrix permeability and likely also structural trends with enhanced fracture permeability. Natural gas along these pathways is preferentially enriched in methane because of selective secondary migration and phase separation processes. The leakage and secondary migration of thermogenic methane to surficial strata is part of an ongoing carbon cycle in which organic carbon in the deep sedimentary basin transforms into methane, and ultimately reaches the near-surface groundwater and atmosphere.

## Abstract (web summary)

As shale and tight gas basins are increasingly used to extract natural gas, understanding how gas migrates is important. Wood and Sanei find that secondary migration in a tight-gas basin leads to up-dip transmission of enriched methane into surficial strata which may leak into groundwater and the atmosphere.

## Graphical abstract

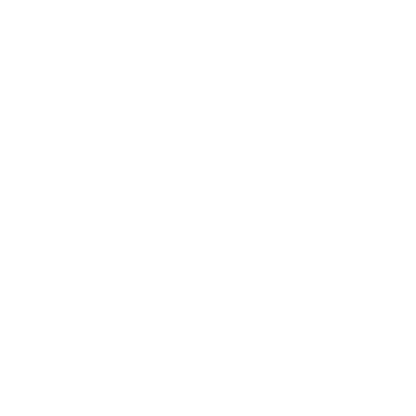

In recent years, shale-gas and tight-gas basins have been increasingly targeted for the application of horizontal drilling and hydraulic fracturing in the extraction of natural gas. The Montney Formation in western Canada is an example of a tight-gas basin that conforms to the definition of an indirect basin-centred gas accumulation^1^, that is, a regionally pervasive, unconventional gas system in which migrated and trapped oil was thermally cracked to gas. Basin-centred gas accumulations go through distinct phases of pressure evolution: normal pressure during early burial, overpressure during deep burial and associated hydrocarbon generation, underpressure during early basin uplift, and return to normal pressure with further uplift^1^,^2^. Depressurization during uplift and erosion of overburden involves temperature reduction and pore volume dilation effects^3^; however, natural leakage of hydrocarbon fluids, primarily gas, is commonly thought to be the dominant factor^3^,^4^,^5^. In this regional study, an extensive set of natural gas composition data from petroleum wells is investigated to show that hydrocarbon fluid loss from the Montney Formation is not uniform, but is instead focused along distinct stratigraphically and structurally controlled pathways within which methane is preferentially enriched during transmission through tight-gas siltstones.

The Lower Triassic Montney Formation is up to 320 m thick and forms part of an overpressured, tight-gas fairway in the Western Canadian Sedimentary Basin^6^,^7^,^8^. The formation is composed mainly of siltstone and was deposited dominantly in lower shoreface to offshore marine environments^8^,^9^,^10^. Turbidite facies were also deposited locally in the succession^11^,^12^. Basin modelling suggests that the Montney tight-gas fairway passed into the thermogenic hydrocarbon generation window ∼80–90 Ma (million years ago)^13^. Present-day total organic carbon, typically in the range from 0.25 to 4.0 wt%, is virtually all in the form of solid bitumen/pyrobitumen^14^,^15^,^16^,^17^. The solid bitumen represents a previous oil phase that partially filled the pore network of Montney siltstones during hydrocarbon charging^16^,^17^. With further burial to maximum depths of 4–7 km (ref. 13) and the accompanying increase in temperature, oil cracked *in situ* to solid bitumen and light hydrocarbon fluids, and significant overpressuring developed. From maximum burial at ∼60 Ma to the present day, the Montney tight-gas fairway underwent considerable depressurization and cooling in response to relaxation of tectonic compression and erosion of 1.4–3.0 km of overburden^13^. In the geographic area of study, which straddles the border between Alberta and British Columbia (Fig. 1), present-day pore pressures in the Montney Formation conform broadly to structural elevation with normally pressured strata in the northeast and overpressured strata located down-dip to the southwest (*cf.* refs 6, 8, 13). The present-day depth of the Montney Formation ranges from 850 m in the northeast to 3,900 m in the southwest, and the transition from normal pressure to overpressure is at a depth of 1,800–2,600 m.

The Montney Formation has a decades-long history of production of conventional hydrocarbon resources, and more recently has emerged as one of the premier unconventional natural gas plays in North America^6^,^7^,^8^. This history of production, under stringent reporting requirements to provincial regulatory authorities, has resulted in publicly available gas composition data for samples of natural gas produced from thousands of petroleum industry wells. Previous studies show that the chemical and isotopic signatures of natural gases can be used to decipher the thermal maturity and migration characteristics of hydrocarbons^18^,^19^,^20^,^21^. Some *n*-alkane signatures have proven to be particularly useful including wetness ratio (Σ C_2_−C_5_/ΣC_1_−C_5_), dryness ratio (C_1_/ΣC_1_−C_5_), the ratio of iso-butane to *n*-butane (iC_4_/nC_4_), and hydrocarbon ratios such as C_1_/C_4_ and C_3_/C_4_ (refs 18, 19, 20, 21). The iC_4_/nC_4_ ratio of natural gas is deemed to be a robust indicator of thermal maturity, whereas wetness and dryness ratios are known to be influenced by both maturity and hydrocarbon migration^19^,^20^,^21^. In this study the terms ‘gas dryness ratio' and ‘normalized methane content' are used interchangeably because the dryness ratio of natural gas is a measure of the methane content normalized to C_1_ through C_5_ components.

The thermal maturity trend of indigenous hydrocarbon fluids is well illustrated by signatures of natural gas produced from the Mississippian Barnett Shale in Texas^21^, a major shale-gas basin with no significant modification by migrated hydrocarbons. A cross-plot of normalized methane content versus iC_4_/nC_4_ ratio shows a distribution with two main maturity segments (Fig. 2). At lower thermal maturity, in the oil to early/mid gas generation windows, the iC_4_/nC_4_ ratio increases with maturity up to a methane (C_1_) content of ∼0.95. At higher thermal maturity, in the late gas generation window (C_1_>0.95), the iC_4_/nC_4_ ratio decreases markedly with further increase in maturity and methane content. The increase in iC_4_/nC_4_ ratio at lower thermal maturity is considered to reflect higher generation rates of iC_4_ than nC_4_ from thermal cracking of kerogen, oil or bitumen^20^. The reversal and decrease in the iC_4_/nC_4_ ratio at higher thermal maturity is considered to reflect the thermal cracking of wet gas and, in particular, the cracking of iC_4_ at a higher rate than nC_4_ (ref. 20).

The huge number of gas analyses publicly available for the Montney Formation provides a unique set of data to investigate the regional distribution and characteristics of natural gas within a single tight-gas formation. Here we investigate the *n*-alkane signatures of natural gas samples from 1,907 petroleum industry wells, in conjunction with vitrinite reflectance measurements of drill-core samples from 31 wells, to interpret the dynamic history of hydrocarbon generation, secondary migration and methane leakage of the Montney tight-gas system. We show that hydrocarbon fluid loss is focused along distinct pathways of secondary migration in response to basin uplift, erosion of overburden and depressurization. Methane content of natural gas is enriched along these stratigraphically and structurally controlled pathways due to selective migration and phase separation processes. Secondary migration leads to up-dip transmission of preferentially enriched methane that leaks into the shallow part of the basin. Leakage of thermogenic methane from the Montney Formation contributes to the global carbon cycle, and is an important example of how organic carbon fixed deeply in sedimentary basins can be unlocked and delivered through dynamic geologic systems to surficial strata and ultimately the atmosphere.

## Results

### Gas composition and thermal maturity

**Figure 2:**
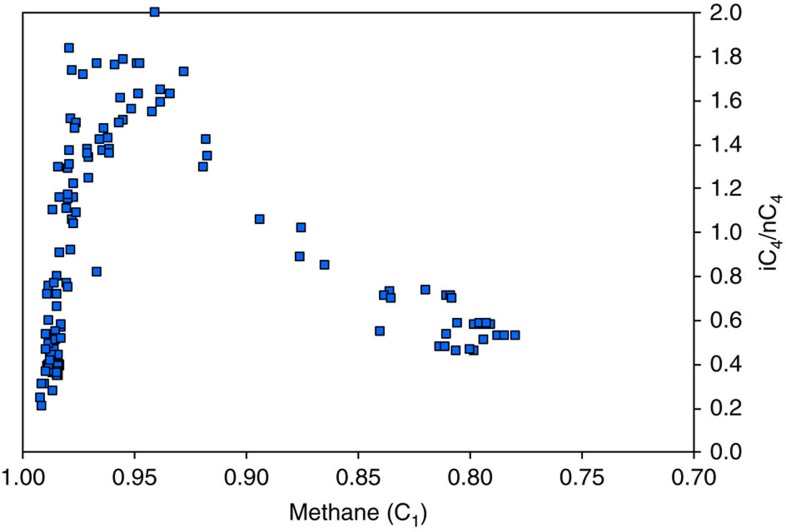
**Cross-plot of normalized methane (C**_**1**_**) content versus iC**_**4**_**/nC**_**4**_ **ratio of natural gas samples from the Barnett Shale in Texas.** The data (blue squares) from 131 wells show a ‘normal' trend at low thermal maturity of iC_4_/nC_4_ ratio increasing with methane content up to 0.95, followed by a ‘rollover', and then a ‘reversal' at high thermal maturity (late gas generation window) of iC_4_/nC_4_ ratio decreasing with methane content. Data from Zumberge *et al*.^21^

**Figure 3:**
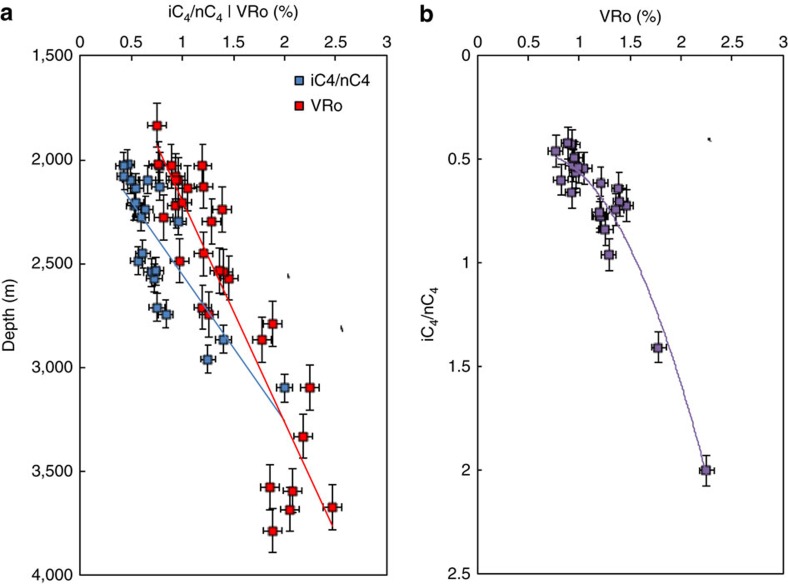
**Cross-plots of iC**_**4**_**/nC**_**4**_ **ratios from natural gas samples and VRo values from drill-core samples from the Montney Formation in western Canada.** VRo signifies vitrinite-equivalent reflectance (%) calculated from bitumen reflectance by use of the Bertrand and Malo^29^ relationship. The data are given in Table 1. (**a**) Depth plot of iC_4_/nC_4_ ratios (blue squares) and VRo values (red squares). Linear trendline for iC_4_/nC_4_ (blue) has *R*^2^ of 0.63 and equation is *y*=701*x*+1,852. Linear trendline for VRo (red) has *R*^2^ of 0.80 and equation is *y*=1,065*x*+1,134. (**b**) Cross-plot of Montney VRo values versus iC_4_/nC_4_ ratios. Best fit polynomial trendline has *R*^2^ of 0.91 and equation is *y*=0.575*x*^2^−0.704*x*+0.692. Error bars on both plots show s.e. The trends of both iC_4_/nC_4_ ratios and VRo values increase with depth (**a**), and these two attributes have a strong positive correlation (**b**), thus supporting the use of the iC_4_/nC_4_ ratio of natural gas samples as a reliable thermal maturity indicator as proposed in previous studies^19^,^20^,^21^.

**Figure 4:**
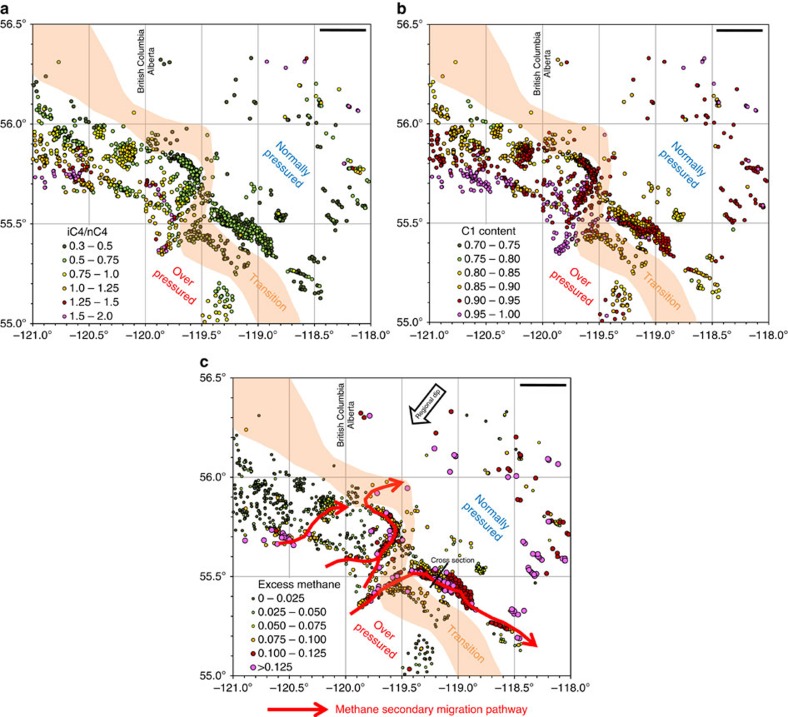
Maps of the study area showing the distribution of Montney natural gas attributes. (**a**) iC_4_/nC_4_ ratio, (**b**) normalized methane content (gas dryness ratio) and (**c**) excess methane content. ‘Excess methane' is defined as the amount of methane greater than expected for indigenous thermal maturity given by the corresponding iC4/nC4 ratio (see plot in Fig. 5). Regional dip is to the southwest as shown by the black arrow in **c**. A transition zone (orange shaded area in all three panels) lies between overpressured section to the southwest and normally pressured section to the northeast. Length of scale bar at top right in each panel is 25 km. Map **a** shows that iC_4_/nC_4_ ratios progressively increase to the southwest in accord with thermal maturity. Map **b** shows a first-order trend of progressively increasing methane content to the southwest, again in agreement with thermal maturity. Second-order curvilinear trends with elevated methane content are also evident. Map **c** shows that regional values of excess methane in the overpressured fairway are dominantly low (black, green dots). High excess methane values (yellow, orange, magenta dots) are focused along curvilinear trends interpreted as methane secondary migration pathways (red arrows). These excess methane trends are high porosity and permeability targets for vertical wells drilled prior to the adoption of horizontal drilling in recent years. Location of cross-section (Fig. 7) through a secondary migration pathway is shown by black line in **c**.

**Figure 5:**
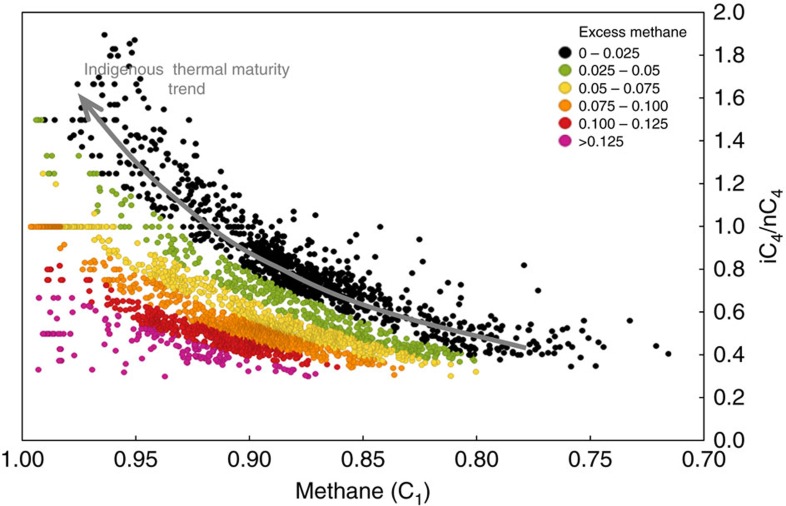
**Cross-plot of normalized methane (C**_**1**_**) content versus iC**_**4**_**/nC**_**4**_ **ratio for natural gas samples from 1,907 petroleum wells drilled into the Montney formation.** Data points are colour-coded by ‘excess methane' defined as the amount of methane greater than the inferred indigenous thermal maturity trend (black dots) at comparable iC_4_/nC_4_ ratio. The excess methane signature (values greater than 0.025 and shown by coloured dots) is interpreted to indicate methane that is introduced to indigenous hydrocarbon fluids by secondary migration. A reversal in the trend of iC_4_/nC_4_ ratios at very high methane contents (>95%), as reported for the Barnett Shale^21^ (Fig. 2), is not evident in the Montney data set.

**Figure 6:**
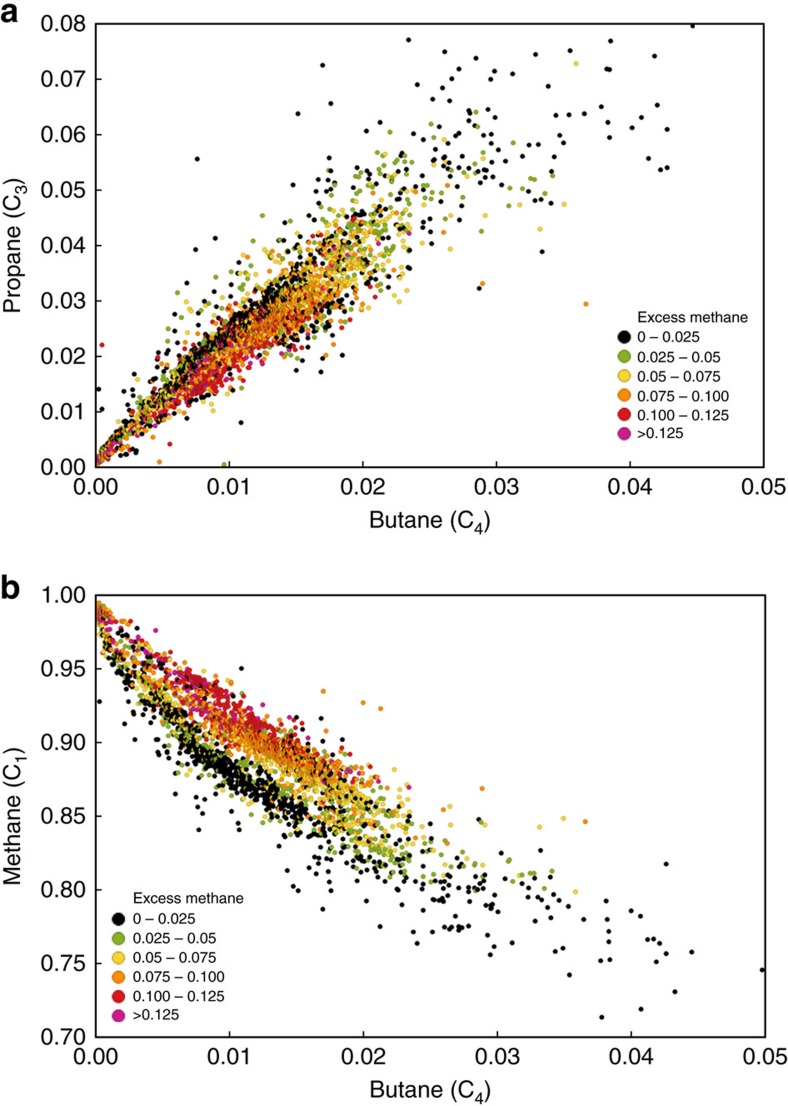
Pixler plots of Montney natural gas composition data. (**a**) Butane (C_4_) versus propane (C_3_) and (**b**) butane (C_4_) versus methane (C_1_). The plots indicate no significant segregation of propane and butane but strong selective segregation of methane with respect to other light *n*-alkanes. Data points are colour-coded by the amount of ‘excess methane' defined as the amount of methane greater than expected for indigenous thermal maturity given by the corresponding iC4/nC4 ratio (see plot in Fig. 5). All values expressed as fractions.

Our study shows that the trends of both iC_4_/nC_4_ ratios and equivalent vitrinite reflectance values increase with depth, and that these two thermal maturity indicators have a strong positive correlation (Fig. 3a,b). Mapped distributions of the key natural gas attributes are shown in Fig. 4a–c. A map of iC_4_/nC_4_ ratios (Fig. 4a) shows progressively increasing values to the southwest in accord with the increase in both depth and thermal maturity towards the Cordilleran foredeep. A map of normalized methane (C_1_) content shows a first-order trend that increases to the southwest, again consistent with thermal maturity (Fig. 4b). The C_1_ map, however, also has second-order trends, oriented orthogonal or oblique to the first-order trend, defined by curvilinear fairways with methane contents higher than expected from the regional first-order C_1_ trend. A cross-plot of normalized methane content versus iC_4_/nC_4_ ratio shows two gradational groups (Fig. 5). The first group (black dots) follows the normal indigenous thermal maturity trend of the Barnett Shale^21^ (*cf.* Fig. 2), and shows a strong positive correlation of iC_4_/nC_4_ ratio with methane content. The second group (coloured dots) is offset to higher methane values than the indigenous hydrocarbons of both the first Montney group and the Barnett Shale trend. The amount of methane greater than expected from the indigenous thermal maturity trend at comparable iC_4_/nC_4_ ratio is expressed as ‘excess methane'; this amount can be as high as 15%. Wells with this excess methane signature are not randomly distributed geographically, but instead are concentrated along the second-order trends on the C_1_ map (Fig. 4b). The second-order C_1_ trends are most clearly evident on a regional map of excess methane (red arrows, Fig. 4c) that was generated using the values from Fig. 5. These excess methane trends are high porosity and permeability targets for vertical wells drilled before the adoption of horizontal drilling in the last decade. From the available well control, the excess methane trends are typically 2–6 km wide and can be mapped from the overpressured Montney section for 10 s km up-dip into the normally pressured Montney section (Fig. 4c). Pixler^18^ plots of C_3_ versus C_4_ and C_1_ versus C_4_, colour-coded by excess methane (Fig. 6a,b), indicate no significant segregation of propane and butane but strong selective segregation of methane with respect to other light *n*-alkanes.

### Stratigraphy and petrophysical properties

**Figure 7:**
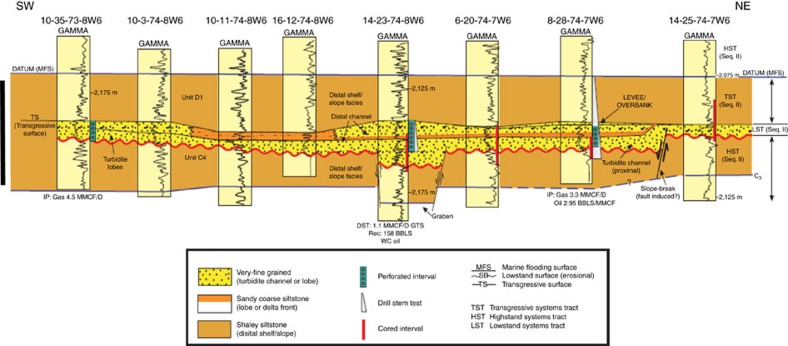
Montney stratigraphic cross-section. Vertical scale bar on left side, 50 m. High-permeability turbidite channel and lobe deposits (yellow dotted pattern) are encased within tighter regional strata (orange shading). Line of section shown on Fig. 5. Well logs shown are natural gamma ray logs. Legend indicates depositional facies, sequence stratigraphy interpretation and well and drill-core information. Used with permission of GCSSEPM^12^. Turbidite deposits with superior matrix permeability are interpreted to form part of a preferential pathway for the secondary migration of methane (Fig. 4c). Turbidite deposition may have been influenced by syn-sedimentary faulting^12^.

Previous studies^11^,^12^ show that the Montney Formation locally contains distinctive turbidite deposits. Stratigraphic cross sections (for example, Fig. 7) and drill-core descriptions document the presence of amalgamated turbidite channel and lobe deposits that can have a combined thickness up to 30 m (ref. 12). The turbidite deposits are composed of coarse-grained siltstone to very fine-grained sandstone and are encased within regional strata composed of tighter, fine-grained siltstone. Our previously published core analysis data sets^8^,^17^ for the overpressured Montney section indicate that turbidite deposits generally have porosity of 6–8% and permeability of 500–5,000 nD, whereas the tighter regional deposits generally have porosity of 1–4% and permeability of 10–100 nD. Turbidite deposits with superior reservoir quality inferred from well logs and drill cores (for example, Fig. 7) are spatially coincident with the excess methane fairways (Fig. 4c) mapped from produced natural gas composition data.

## Discussion

The use of the iC_4_/nC_4_ ratio of natural gas samples as a reliable thermal maturity indicator as proposed in previous studies^19^,^20^,^21^ is supported by the results of our Montney study: we find that the trends of both equivalent vitrinite reflectance values (the most widely accepted indicator of thermal maturity) and iC_4_/nC_4_ ratios increase with depth, and that these two attributes have a strong positive correlation (Fig. 3a,b). For a basin-centred gas accumulation in which the distribution of hydrocarbon fluids is solely a function of thermal maturity, the iC_4_/nC_4_ ratio should therefore be expected to closely follow the dryness of the natural gas. This is the case for the Barnett Shale that, on a cross-plot of normalized methane content (gas dryness ratio) versus iC_4_/nC_4_ ratio (Fig. 2), shows a low thermal maturity trend of iC_4_/nC_4_ ratio increasing with methane content, followed by a ‘rollover' and then a reversed trend at high thermal maturity (late gas generation window) of iC_4_/nC_4_ ratio decreasing with methane content. A comparable Montney cross-plot of normalized methane content versus iC_4_/nC_4_ ratio (Fig. 5) shows a more complex distribution. A portion of the data (black dots, Fig. 5) matches the low thermal maturity trend of the Barnett Shale, representing indigenous hydrocarbon fluids that are distributed in accord with original thermal maturity. Many data points on the Montney cross-plot, however, lie to the left of the indigenous thermal maturity trend. These data points (coloured dots, Fig. 5) have an ‘excess methane' signature, indicating that the maturity-controlled distribution of indigenous hydrocarbon fluids is modified by the introduction of methane. This introduced methane reflects secondary migration in response to basin uplift, erosion of overburden and depressurization. A comparison of the normalized methane content versus iC_4_/nC_4_ ratio cross-plots of the Barnett (Fig. 2) and the Montney (Fig. 5) also shows that the reversed trend at high thermal maturity in the Barnett data is not evident in the Montney data. Reversed iC_4_/nC_4_ trends at high thermal maturity are thought to result from the cracking of wet gas in an essentially closed system^20^. The fact that the dominant lithology of the Montney tight-gas fairway is coarse-grained siltstone suggests a more permeable and open system than in true shale systems like the Barnett. The more permeable Montney system allows the secondary migration of methane to overprint an original maturity-controlled distribution of hydrocarbons similar to that seen in the closed Barnett shale system.

The geographic distribution of wells with an excess methane signature (Fig. 4c) indicates that secondary migration of methane through the Montney basin-entred gas accumulation is not uniform, but is focused along distinct curvilinear pathways. Integration of drill-core studies^8^,^11^,^12^,^17^, subsurface mapping (Fig. 4a–c) and seismic interpretation suggests that these secondary migration pathways commonly follow stratigraphic trends with superior matrix permeability (Fig. 7). The observation of natural fractures in Montney drill cores in this study and by others^22^ opens the possibility that structural trends with locally enhanced fracture permeability might also contribute to the secondary migration pathways, but further quantitative data on fracture distribution is required to test this interpretation. Hydrocarbon molecules (primarily methane) in these secondary migration pathways are driven up-dip by regional pressure gradient and responded to the dynamic, non-equilibrium conditions of pore pressure and *in situ* stress reduction during 50–60 million years of basin uplift. Accelerated leakage of methane from these migration pathways contributed to the overall depressurization of the Montney basin-centred gas accumulation, and also led to gas charging of conventional traps within Montney turbidite channel reservoirs located closely up-dip (Fig. 4c). The enormous volume of overpressured, ultra-dry gas located down-dip to the southwest (Fig. 4b and Kuppe *et al*.^6^) provides a practically inexhaustible source of methane to supply the secondary gas migration process. Secondary migration of gas driven up-dip through ‘high-permeability streaks' and natural fractures by differential pressure has also been suggested for the Montney Formation further to the southeast in the tight-gas fairway^6^.

The segregation of methane with respect to other light hydrocarbon molecules, as evinced in Pixler^18^ plots of C_3_ versus C_4_ and C_1_ versus C_4_ (Fig. 6a,b) in addition to the normalized methane content versus iC_4_/nC_4_ cross-plot (Fig. 5), indicates that the dominant mode of secondary migration in response to differential pressure is the selective molecular transmission of primarily methane rather than the mass transport of natural gas composed of heavier *n*-alkanes as well as methane. The regionally continuous fluid phase in the overpressured Montney basin-centred gas accumulation is natural gas not water^6^,^8^; thus, gravitational fractionation due solely to buoyancy segregation of water and hydrocarbons does not satisfactorily explain excess methane along the curvilinear migration pathways (although this is an important mechanism within conventional hydrocarbon traps located up-dip). An additional consideration is the response of hydrocarbon fluids to changed thermodynamic conditions: progressive decrease in temperature and especially pressure during basin uplift may have led to an original supercritical, single-phase hydrocarbon fluid eventually separating into gas and liquid phases^23^. The addition of hydrocarbon liquids to formation water already in the pore network may then have been sufficient to promote gravitational fractionation of gas and liquids due to buoyancy segregation. Gas exsolved in this manner is typically enriched in light *n*-alkanes^23^, particularly methane. The lower density and greater mobility of methane compared with other hydrocarbon molecules could then have led to selective secondary migration of methane-enriched dry gas along pathways of enhanced matrix and fracture permeability in response to buoyancy as well as regional pressure gradient.

The observed segregation of methane might also be compatible with some form of geochromatography^24^ involving gas–solid and/or gas–liquid fractionation with natural gas as the mobile phase. Minerals and organic matter would form a stationary solid phase, whereas capillary-bound water and clay-bound water would form a stationary liquid phase. One possible mechanism of gas–solid fractionation in the Montney is size-exclusion chromatography^24^,^25^, whereby the smallest hydrocarbon molecule, methane, is able to preferentially pass through the extremely narrow throat constrictions of the complex pore network^17^,^26^,^27^, but larger hydrocarbon molecules are mostly excluded. Effective diameters of the C_1_ through C_4_ molecules are all similar at ∼0.4 nm, whereas effective length increases from 0.40 nm for C_1_ to 0.82 nm for C_4_. The limited range of these molecular dimensions seem to offer limited scope for natural size-exclusion chromatography. Furthermore, these molecular dimensions are much smaller than the typical aperture size of pore throats in Montney samples determined from mercury injection capillary pressure measurements, nitrogen adsorption data and focused ion beam/scanning electron imaging. Pore throat aperture is generally in the range of 3.5–200 nm, although apertures as small as 0.6 nm (similar to the molecular dimensions of the light *n*-alkanes) are reported^14^,^17^,^26^,^27^. We thus consider that size-exclusion chromatography likely had limited influence on methane segregation in the Montney during secondary migration.

Another potential mechanism of gas–solid fractionation is adsorption chromatography^24^,^25^, whereby migrating hydrocarbon molecules are differentially adsorbed on mineral and especially organic matter substrates. This mechanism can result in selective retention of hydrocarbon components and thus the differential retardation of the migration rate of hydrocarbons^25^. For mobile light *n*-alkanes and stationary organic matter, as is the case in the Montney tight-gas fairway, methane has the lowest sorption affinity and would be expected to have the shortest retention time and conversely the fastest rate of migration.

Furthermore, stratigraphic differences in total sorptive capacity might have influenced the migration rates of light *n*-alkanes through the Montney Formation under the non-equilibrium conditions induced by basin uplift and progressive depressurization. Secondary migration pathways (Fig. 4c) commonly follow distinct stratigraphic trends with superior matrix quality (Fig. 7). Rocks along these stratigraphic trends generally have lower organic matter content (dominantly solid bitumen) than adjacent regional strata^16^,^17^, and therefore likely have lower total sorptive capacity. Low total sorptive capacity along these stratigraphic trends would have led to reduced sorptive retention time and further acceleration of methane migration.

In summary, our regional study of produced natural gas from 1,907 petroleum wells drilled into the Lower Triassic Montney Formation in western Canada reveals that hydrocarbon fluid loss from a basin-centred gas accumulation is focused along distinct curvilinear pathways of secondary migration in response to basin uplift, erosion of overburden and depressurization. The secondary migration pathways follow stratigraphic trends with superior matrix quality and likely also structural trends with enhanced fracture permeability. The methane content of natural gas is preferentially enriched along these pathways due to various selective migration and phase separation processes. The enormous volume of overpressured, ultra-dry gas located down-dip to the southwest (Fig. 4b and Kuppe *et al*.^6^) provides an almost inexhaustible supply of methane to feed the secondary gas migration process.

The selective secondary migration within the overpressured Montney tight-gas system leads to regional, up-dip transmission of preferentially enriched methane, a powerful greenhouse gas, which leaks into the normally pressured, shallow part of the basin and ultimately reaches the near-surface groundwater and atmosphere. The leakage of thermogenic methane from the Montney Formation contributes to the ongoing global carbon cycle, and is an important example of how the immense mass of organic carbon fixed deeply in sedimentary basins can be unlocked and transported up-dip, through open and dynamic systems^28^. This process not only strongly influences the spatial distribution of hydrocarbon resources within the Montney tight-gas basin, but also leads to the ongoing leakage of methane to the atmosphere and globally significant natural greenhouse gas emissions.

## Methods

### Vitrinite-equivalent reflectance

Organic petrology studies are performed on drill-core samples from 31 wells (Table 1). Polished blocks are made with a cold-setting epoxy-resin mixture. The resulting sample pellets are ground and polished in final preparation for microscopy, which was carried out using an incident light Zeiss Axioimager II microscope system equipped with fluorescent light sources and the Diskus–Fossil system for reflectance measurements. Random reflectance (Ro) measurements are conducted under oil immersion by use of an ultra-fine pixel size (0.3 μm) probe. Whenever possible, greater than 100 measurements are made on each sample to construct a robust reflectance histogram. In some organically lean samples, the number of measurements is lower because only reliable organic particles with well-polished surfaces are measured. The measured bitumen reflectance (BRo) values are converted to the equivalent vitrinite (random) reflectance values (VRo) using the Bertrand and Malo^29^ relationship (VRo=(BRo+0.03)/0.96).

### Natural gas compositional data

We compiled gas composition data for samples of natural gas produced from 1,907 petroleum industry wells drilled into the Montney Formation. All these routine gas analyses are publicly available from provincial regulatory authorities, the British Columbia Oil and Gas Commission and the Alberta Energy Regulator. For this study, gas analyses were accessed by use of third-party commercial software (Geofluids version 3.5, IHS Energy). All wells with Montney gas analyses from the study area at the time of data access (29 July 2013) are included in this study. These gas analyses are performed at commercial laboratories by use of the gas chromatography method following standards published by the Gas Processors Association for routine gas analysis (for example, GPA-2261) or extended gas analysis (for example, GPA-2286). The compiled gas composition data typically report nitrogen, carbon dioxide, hydrogen, helium and hydrogen sulfide in addition to hydrocarbons. Gas compositions are normalized to just the methane through pentane (C_1_ to C_5_) hydrocarbon components for two main reasons. First, wellbore operations such as hydraulic fracture stimulations commonly introduce nitrogen and carbon dioxide to indigenous natural gases. Second, the hydrocarbon components are variously reported out from C_5_ to C_10_, and normalization of just the C_1_ to C_5_ components provides a basis for consistent analysis of the data. For ease of graphical presentation, gas analyses with normalized methane content less than 70% are omitted from the final data set shown here (Figs 5 and 6a,b). Gas samples with iC_4_/nC_4_ ratio greater than 2.0 are also omitted because these samples typically have extremely low nC_4_ and iC_4_ contents, which introduce unacceptable error in calculating their ratio. The normalized gas composition analyses are investigated using simple mapping (Fig. 4) and cross-plotting (Figs 5 and 6a,b) techniques. We investigated the use of the iC_4_/nC_4_ ratio as a thermal maturity indicator by selecting natural gas samples from wells offsetting those with vitrinite-equivalent reflectance measurements of drill-core samples (Table 1). The distance of offset is generally less than 4 km but up to 9 km in sparsely drilled areas.

### Data availability

The data that support the findings of this study are available from the corresponding author on request.

## Additional information

**How to cite this article:** Wood, J. M. & Sanei, H. Secondary migration and leakage of methane from a major tight-gas system. *Nat. Commun.* **7,** 13614 doi: 10.1038/ncomms13614 (2016).

**Publisher's note**: Springer Nature remains neutral with regard to jurisdictional claims in published maps and institutional affiliations.

## Figures and Tables

**Figure 1:**
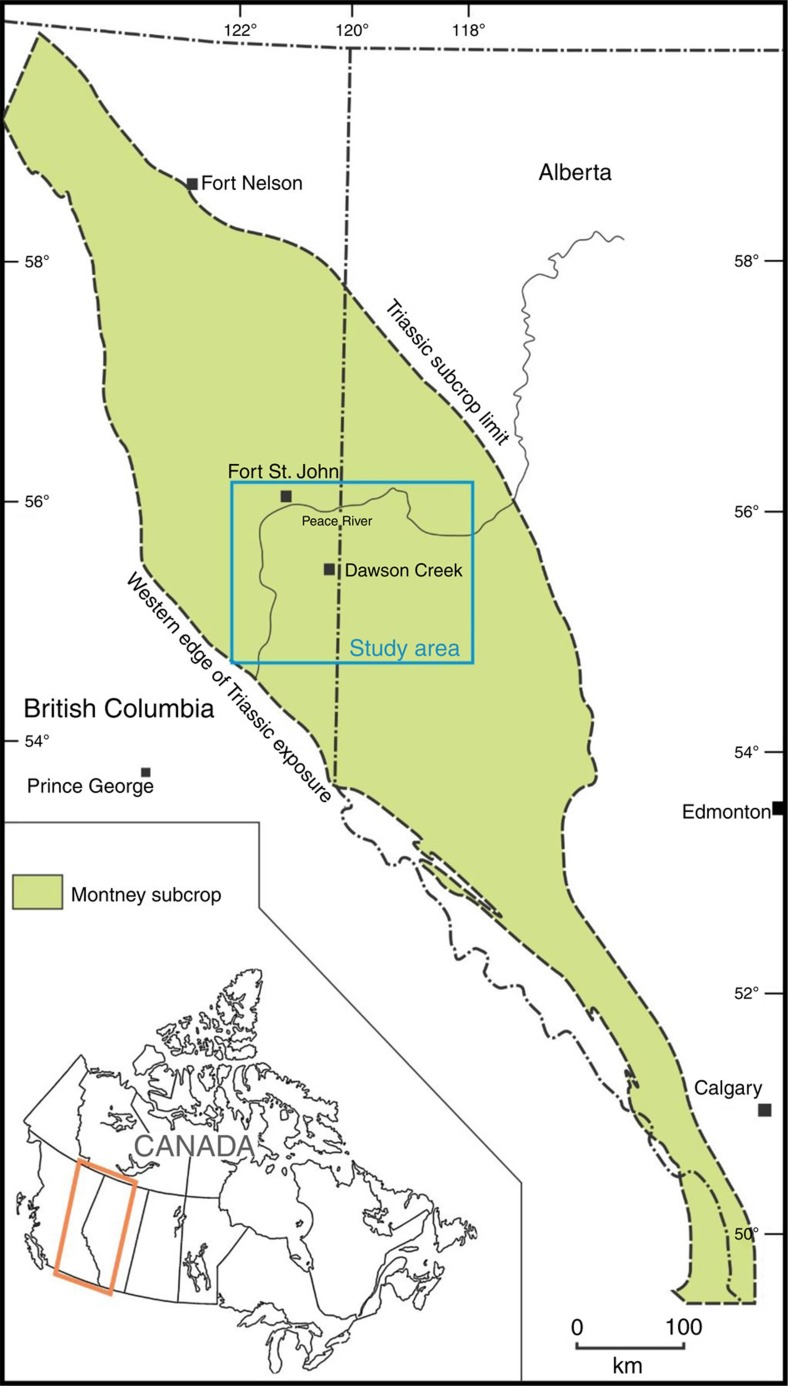
Map showing location of the study area in western Canada. Green shaded area shows extent of Montney Formation and age-equivalent strata. Blue box shows study area. Scale bar, 100 km.

**Table 1:** Vitrinite-equivalent reflectance (VRo %) data from drill-core samples and iC_4_/nC_4_ ratios of natural gas sample from the Montney Formation of western Canada.

**Unique well identifier**	**Latitude**	**Longitude**	**Depth, m (mean)**	**VRo, %**		**iC4/nC4**	
				**Average**	**n**	**Average**	**n**
100/01-14-075-11W6/00	55.49381	−119.58321	2,486	0.97	1	0.5647	9
100/01-20-073-13W6/00	55.33126	−119.94986	2,960	n/a	0	1.2500	2
100/01-32-070-09W6/00	55.09879	−119.32408	2,569	1.46	5	0.7228	10
100/01-36-079-15W6/00	55.88397	−120.19444	2,031	1.20	3	0.7767	18
100/03-21-080-17W6/00	55.94528	−120.59961	2,239	1.39	7	0.6388	20
100/04-19-077-10W6/00	55.68144	−119.54587	2,136	1.05	1	0.5418	12
100/06-14-078-11W6/00	55.75887	−119.59732	2,223	0.94	1	0.5294	10
100/06-12-079-12W6/00	55.83161	−119.73766	2,021	0.77	3	0.4598	21
100/07-34-078-11W6/00	55.80268	−119.61260	2,096	0.96	2	0.4932	8
102/12-35-079-17W6/00	55.89137	−120.55155	2,127	1.21	4	0.7766	19
100/14-36-078-12W6/00	55.80677	−119.72579	2,079	0.93	7	0.4309	11
100/13-12-078-11W6/00	55.75049	−119.57406	2,205	1.00	6	0.5396	23
100/13-22-070-08W6/00	55.06791	−119.10428	2,453	1.21	3	0.6150	5
100/14-09-077-11W6/00	55.66170	−119.64741	2,277	0.82	1	0.5986	17
100/14-13-078-16W6/00	55.76529	−120.34276	2,295	1.29	2	0.9579	2
100/15-08-081-12W6/00	56.01121	−119.83558	1,833	0.75	1	n/a	0
100/15-13-080-17W6/00	55.94090	−120.51625	2,098	0.93	6	0.6590	11
100/16-10-079-12W6/00	55.83804	−119.77794	2,031	0.89	2	0.4210	7
100/16-35-078-21W6/00	55.80729	−121.13412	2,864	1.78	4	1.4055	8
200/A-036-G-093-P-01/00	55.11066	−120.18741	3,673	2.47	2	n/a	0
200/A-040-J-093-P-01/00	55.19137	−120.24009	3,593	2.08	4	n/a	0
200/B-030-H-093-P-09/00	55.60184	−120.12610	2,541	1.40	2	0.7003	19
200/B-032-G-093-P-09/00	55.60836	−120.14809	2,710	1.20	1	0.7534	8
200/A-034-A-093-P-11/00	55.52559	−121.03916	3,785	1.88	4	n/a	0
200/C-010-E-093-P-10/00	55.58619	−120.99058	3,577	1.86	13	n/a	0
200/C-015-L-093-P-07/00	55.42514	−120.93091	3,682	2.05	6	n/a	0
200/C-025-G-093-P-09/00	55.60443	−120.18743	2,743	1.26	3	0.8364	9
200/C-081-J-093-P-07/00	55.48941	−120.63387	3,331	2.18	2	n/a	0
200/D-021-G-093-P-09/00	55.60452	−120.13237	2,534	1.36	3	0.7412	6
200/D-065-G-093-P-08/00	55.38768	−120.18225	3,098	2.25	6	2.0000	2
200/D-085-A-093-P-10/00	55.57266	−120.55563	2,790	1.88	4	n/a	0

iC4/nC4, ratio of iso-butane to *n*-butane; n/a, sample of produced gas not available within acceptable distance of drill-core sample with VRo measurement; VRo, equivalent vitrinite reflectance.

VRo (%) calculated from bitumen reflectance by use of the Bertrand and Malo^29^ relationship.

Natural gas samples are from wells generally within a 4 km radius (maximum 9 km) of wells with VRo measurements of drill-core samples. *n*=number of samples.

## References

[b1] Law B. E. Basin-centered gas systems. Am. Assoc. Pet. Geol. Bull. 86, 1891–1919 (2002).

[b2] Law B. E. & Dickinson W. W. Conceptual model for origin of abnormally pressured gas accumulations in low-permeability reservoirs. Am. Assoc. Pet. Geol. Bull. 69, 1295–1304 (1985).

[b3] Meissner F. F. & Thomasson M. R. Exploration opportunities in the Greater Rocky Mountain Region, U.S.A. AAPG Memoir 74, 201–239 (2001).

[b4] Burnie S. W. Sr. . Experimental and empirical observations supporting a capillary model involving gas generation, migration, and seal leakage for the origin and occurrence of regional gasifers. AAPG Hedberg Series 3, 29–48 (2008).

[b5] Hao F. . Factors controlling petroleum accumulation and leakage in overpressured reservoirs. Am. Assoc. Pet. Geol. Bull. 99, 831–858 (2015).

[b6] Kuppe F. C., Nevokshonoff G. & Haysom S. in SPE Canadian Unconventional Resources Conference, 30 October–1 November (Society of Petroleum Engineers, Calgary, Canada, (2012).

[b7] National Energy Board. The Ultimate Potential for Unconventional Petroleum from the Montney Formation of British Columbia and Alberta. (National Energy Board, Briefing Note (2013).

[b8] Wood J. Water distribution in the Montney tight gas play of the western Canadian sedimentary basin: significance for resource evaluation. SPE Reserv. Eval. Eng. 16, 290–302 (2013).

[b9] Edwards D. E. . in Geological Atlas of the Western Canada Sedimentary Basin Ch. 16, 257–275Canadian Society of Petroleum Geologists & Alberta Research Council, (1994).

[b10] Davies G. R., Moslow T. F. & Sherwin M. D. The Lower Triassic Montney Formation, west-central Alberta. Bull. Can. Pet. Geol. 45, 474–505 (1997).

[b11] Moslow T. F. & Davies G. R. Turbidite reservoir facies in the Lower Triassic Montney Formation, west-central Alberta. Bull. Can. Pet. Geol. 45, 507–536 (1997).

[b12] Moslow T. F. in *Deep-water Reservoirs of the World, Conference Proceedings* (eds Weimer P.. 686–713Gulf Coast SEPM (2000).

[b13] Ness S. M. *The Application of Basin Analysis to the Triassic Succession, Alberta Basin: an Investigation of Burial and Thermal History and Evolution of Hydrocarbons in Triassic Rocks.* Unpublished M.Sc. Thesis, Univ. Calgary (2001).

[b14] Chalmers G. R. & Bustin R. M. Geological evaluation of Halfway–Doig–Montney hybrid gas shale–tight gas reservoir, northeastern British Columbia. Mar. Petrol. Geol. 38, 53–72 (2012).

[b15] Freeman M. J. *Lithological, Diagenetic, and Organic Controls on Reservoir Quality in the Lower Triassic Montney Formation, Pouce Coupe, Alberta.* Unpublished M.Sc. Thesis, Univ. Calgary (2012).

[b16] Sanei H. . Characterization of organic matter fractions in an unconventional tight gas siltstone reservoir. Int. J. Coal Geol. 150, 296–305 (2015).

[b17] Wood J. M. . Solid bitumen as a determinant of reservoir quality in an unconventional tight gas siltstone play. Int. J. Coal Geol. 150, 287–295 (2015).

[b18] Pixler B. O. Formation evaluation by analysis of hydrocarbon ratios. J. Pet. Technol. 21, 665–670 (1969).

[b19] Prinzhofer A., Mello M. R. & Takaki T. Geochemical characterization of natural gas: a physical multivariable approach and its applications in maturity and migration estimates. Am. Assoc. Pet. Geol. Bull. 84, 1152–1172 (2000).

[b20] Hao F. & Zou H. Cause of shale gas geochemical anomalies and mechanisms for gas enrichment and depletion in high-maturity shales. Mar. Petrol. Geol. 44, 1–12 (2013).

[b21] Zumberge J., Ferworn K. & Brown S. Isotopic reversal (‘rollover') in shale gases produced from the Mississippian Barnett and Fayetteville formations. Mar. Petrol. Geol. 31, 43–52 (2012).

[b22] Davies G. R. . in SPE/AAPG/SEG Unconventional Resources Technology Conference, 25–27August (Society of Petroleum Engineers, Denver, CO, USA, (2014).

[b23] di Primio R., Dieckmann V. & Mills N. PVT and phase behaviour analysis in petroleum exploration. Org. Geochem. 29, 207–222 (1998).

[b24] Krooss B. M., Brothers L. & Engel M. H. Geochromatography in petroleum migration: a review. Geol. Soc. Spec. Pub. 59, 149–163 (1991).

[b25] Matthews M. D. Migration — a view from the top. AAPG Memoir 66, 139–155 (1996).

[b26] Clarkson C. R. . Nanopore-structure analysis and permeability predictions for a tight gas siltstone reservoir by use of low-pressure adsorption and mercury-intrusion techniques. SPE Reserv. Eval. Eng. 15, 648–661 (2012).

[b27] Ghanizadeh A. . Petrophysical and geomechanical characteristics of Canadian tight oil and liquid-rich gas reservoirs: I. Pore network and permeability characterization. Fuel 153, 664–681 (2015).

[b28] Kroeger K. F., di Primio R. & Horsfield B. Atmospheric methane from organic carbon mobilization in sedimentary basins—the sleeping giant? Earth Sci. Rev. 107, 423–442 (2011).

[b29] Bertrand R. & Malo M. Source rock analysis, thermal maturation and hydrocarbon generation in the Siluro-Devonian rocks of the Gaspe Belt basin, Canada. Bull. Can. Pet. Geol. 49, 238–261 (2001).

